# A Monograph on Excisions

**Published:** 1840

**Authors:** J. F. Malgaigne


### Selections from American and Foreign Journals.

---

*A Monograph on Excisions*, translated from the French of J. F. Malgaigne, for the Western Journal of Medicine and Surgery, by Benjamin Dennis, M. D., of Cincinnati.

### General observations on Excisions.

Under this title we shall comprehend not only the ablation of the articular extremities of the bones, but also the excision of the long pieces in their continuity, and finally the entire extirpation of certain bones, without amputation of the soft parts.

Before we proceed further, let us lay down some general precepts.

1. It is necessary before commencing the operation, to have a clear perception of the anatomical derangements, which render it much more difficult on the living than on the dead subject; these are, the swelling of the bones; sometimes their union; induration and engorgement of the soft parts, and the difficulty of recognizing the nerves and blood-vessels, which must be carefully avoided.

Let the operator, says the younger Moreau, proceed with composure and tranquillity of mind: surgery requires prudence and is devoid of timidity.

2. Besides the ordinary dressing apparatus, we must have at hand, the excision forceps, a gouge, a mallet, and saws of different sizes and forms, so that we may not be taken unawares should it become necessary to change any of them during the operation.

3. An assistant should be in readiness, with a good supply of water and fine sponges, to wipe away the blood, so that the color of the bones may be recognized.

4. In making the external incisions, our object should be, to procure a sufficient and commodious passage to the bone, without unnecessarily injuring the muscles and tendons.

When we wish to remove completely a long bone, should a simple longitudinal incision not suffice, we may, as a general proposition, add a small perpendicular incision to each extremity of the first, in order to obtain a quadrilateral flap.

5. It is necessary to avoid carefully the division of the nervous, venous, and arterial trunks.

6. The bone being exposed, we should probe it with the gouge or a pointed stylet for the purpose of ascertaining the depth of the caries.

7. Before employing the saw, we should carefully protect the soft parts from the action of that instrument, by means of compresses or by passing the handle of a bistoury between them and the saw.

8. The first law is to remove completely all that portion of the bone which is carious. To this all the others are subordinate.

9. The second law is to save as far as possible the tendons and muscular attachments. Nevertheless the carious portion of the bone should be entirely removed.

The slightest layer of compact substance will be sufficient to preserve the attachment of the muscles.

10. When an articular surface is composed of several bones, we should generally cut off the pieces at the same level, in order to avoid obliquity of the section, which would force the limb to one side or the other. This rule is particularly important in relation to the articulations of the wrist and ancle.

11. If the excision is practised in the continuity of a long bone, or if we wish to extract the bone entirely, we should preserve as much of the periosteum as the malady will permit. In children it furnishes the matter for a new bone, and in adults it serves as the basis of a fibrous tissue, which replaces to a certain extent, the old bone.

12. Having performed the operation, the lips of the wound are to be brought into contact, and retained in that state by adhesive strips and the interrupted suture.

13. In operating on the articulations of the inferior extremities, the bones should be brought together, and the limb extended; but when the operation is performed on the superior extremities, the limb should be semi-flexed, leaving the bones slightly separated, in order to obtain, if possible, an artificial joint.

### OF EXCISIONS OF PARTICULAR BONES.

We shall treat successively of partial or complete excisions of the bones of the superior extremities then of those of the inferior, and finally of the bones of the trunk.

### ART. I.—EXCISION OF THE BONES OF THE SUPERIOR EXTREMITIES.

### 1. *Excision of the Metacarpo-Phalangeal Articulation.*

In performing this operation the condition of the case may render necessary the removal either of the head of the metacarpal bone, the extremity of the phalanx or both together.

The operation should be commenced by making an oblique incision, beginning at the middle of the dorsal face of the metacarpal bone, about half an inch beyond the point where we wish to apply the saw, and terminating at the commissure of the finger; a second incision should be made commencing at the same point and ending at the other commissure; circumscribing thus a flap in the shape of the letter v with its base downwards. This flap should be dissected up and turned backwards; the tendon of the extensor, thus brought into view, removed; and the interosseous muscles on each side of the bone, detached. The articulation should then be opened; and the lateral and anterior ligaments cautiously divided, avoiding all injury to the flexor tendons. After which the phalanx must be dislocated backwards; and having carefully freed the diseased portion of bone from the soft parts, and introduced behind it a piece of wood or pasteboard, the operation should be finished by the application of the saw. The excision of the head of the metacarpal bone is conducted in the same manner.

If the excision is to be practised on the articulation of the thumb, the index, or the little finger, the flap should be made on the free side of the finger, with its base above or below as the state of the case may require. By adopting this method, we shall not be compelled to push the tendon of the extensor muscle much out of the way on account of the saw.

M. Bobe once excised the head of the first phalanx of the thumb, in a case of irreducible luxation; this circumstance removed many of the difficulties attending the operation.

### II.—EXTRACTION OF THE FIRST PHALANX.

Although, this operation has never been attempted, nor even proposed, yet it appears to be sometimes indicated on the thumb. M. Velpeau has seen the smaller phalanx of this finger preserve its movements after the extraction of fragments of the first phalanx affected with necrosis.

In conducting this operation, we should make the incision on the radial side of the thumb; remove the soft parts cautiously in order to save as much of the periosteum as possible; tenderly destroy the metacarpal articulation; and dislocate the bone outwards. The rest will be easy.

### III.—Extraction of the metacarpal bone.

Proposed by M. Troccon in 1816, and afterwards performed with success, by M. Roux and M. Blandin.

This operation is performed by making an incision along the radial side of the bone, extending about half an inch beyond each of its articulations; the skin and the extensor tendon of its dorsal face and the muscles of its palmar face should be cautiously detached. An assistant should forcibly separate the lips of the wound; whilst the surgeon carries the point of the bistoury on the external side of the carpal articulation; divides the tendon of the abductor longus, which is attached to the metacarpal bone; and destroys the articulation. The bone should then be luxated outwards; and the bistoury carried along its internal face, in order to separate the flesh from it completely. Finally its articulation with the phalanx should be destroyed, by dividing successively the internal, external, and anterior lateral ligaments.

The radial artery can be easily avoided; and the rest secured by the application of the ligature. The lips of the wound should be retained in apposition by the aid of lint or compresses; and union by the first intention induced. The palm of the hand should be so dressed, that the thumb may be maintained in its natural position. After recovery, the finger is contracted and at first incapable of any use; however, it will gradually acquire all its natural movements.

If the first incision should not suffice, another should be added to each of its extremities, according to the general method indicated.

### IV.—Extraction of the other metacarpal bones.

The metacarpal bone of the index finger, can be easily extracted by means of an incision on its external surface: and that of the little finger by the aid of an incision on its external side. But the probable shortening of these fingers will leave a deformity as great perhaps, as that produced by their amputation, and will, without doubt, lessen the general force of the hand.

A different method should be adopted for the extraction of the metacarpal bones corresponding to the middle and ring fingers. The incision should be made along the dorsal face of the bone, by the side of the extensor tendon; (which must be carefully protected) and the disarticulation begun at the joint of the phalanx. These two fingers can be easily maintained in their natural positions, by their connection with the neighboring ones.

### V.—Extirpation of the bones of the carpus.

Sir A. Cooper extracted with success the os scaphoides in a case of luxation of that bone, occasioned by pressure from a wool-carding machine. He lays down this principle, that, when, by the effect of an analogous cause, one or two bones of the carpus are displaced, extraction may be practised; but, if the injury be greater, amputation is necessary.

### VI.—Excision of the articulation of the wrist.

*Method of M. Roux.*—M. Roux makes, along the antero-external part of the radius and antero-internal margin of the ulna, without interfering with the vessels and nerves of the fore-arm, two longitudinal incisions terminating inferiorly on a level with the joint of the wrist; also, two transverse incisions extending backwards from the inferior part of the fore-arm, to the sides of the bundle of the extensor tendons at the posterior surface of the articulation. Two flaps in the shape of the letter V were thus obtained, which he dissected up, carefully avoiding the tendons which play in the dorsal grooves of the bones; these were then held out of the way by a compress, spatula, or piece of paste-board; he then applied the saw to the ulna; an assistant forcibly bent the hand outwards in order to aid the operator in separating the interosseous attachments with the radius and carpus. Finally, the hand was forced inwards, which rendered it easy, either to saw the radius before or after the disarticulation. The wound thus made was large enough to expose the surface of the carpal bones, wnich enabled him to remove as many of them as the necessity of the case required.

M. Dubled made a single longitudinal incision on the side of each bone, commencing always with the ulna; forced the hand inwards; destroyed the ulnar articulation; and dislocated the bone, before excising it. He then passed to the radius which he excised in the same manner.

M. Velpeau on the contrary, completely unites the longitudinal incisions, by means of a transverse one on the back of the wrist, in order to procure a quadrilateral flap with its base downwards; which he dissects up and turns back on the hand. Having protected the tendons as much as possible, he detaches the flesh from the palmar face of the bones; passes under the latter a protecting plate; and saws both bones at once. He then turns them back, separating them from the bones of the carpus.

*Appreciation.*—There is this in common in these three methods that one may be easily converted into the other during the same operation. It is best, however, to commence with the simple incisions of M. Dubled, as they certainly afford the happiest results. If the operation be difficult, we may add to these the small transverse incisions of M. Roux; and when the case presents unusual embarrassments we may unite the two smaller flaps into one, by a complete transverse incision after the manner of M. Velpeau. It is a matter of indifference whether the flap has its base above or below.

It is sometimes necessary, in fractures of the radius accompanied with luxation of the ulna, to excise the extremity of the latter. The operation in this case is generally facilitated by the displacement; but we must be careful that the ulna retains the same length with the fractured radius, otherwise the hand will incline to the side of the shorter bone.

### VII.—Extirpation of the Radius.

This operation was successfully practiced in 1825 by Dr. Butt of Virginia.

The arm is to be placed in a semi-flexed position, and a longitudinal incision made along the antero-external side of the radius, down to the bone. The integuments should then be detached, and protected from injury by passing behind the bone a canula or the handle of the scalpel; after which the bone should be separated with the ordinary, or chain saw.

It will then be necessary only, to separate each of the fragments from the flesh; and sever them from their articulations, carefully avoiding, in the mean time, the blood-vessels and nerves.

In this operation particularly, the terminal incisions, advised by M. Roux for the wrist, and by M. Moreau for the elbow, would be of evident utility.

### VIII.—Excision of the articulation of the elbow.

We will barely mention here that the method of Park consisted in making, along the middle of the posterior surface of the arm, a longitudinal incision, which was, when necessary, converted into a crucial one. This method is now entirely abandoned.

*Method of Moreau.*—In performing the operation of Moreau, the patient should be placed on his abdomen, upon a table covered with a matrass, in such a manner, that the arm resting upon the side of the table presents to the operator the posterior and internal part of the articulation; and the tourniquet applied round the superior third of the arm, and confided to an intelligent assistant.

The arm being half flexed; the surgeon with a dissecting-scalpel or a bistoury, makes an incision parallel with the sharp edge or spine of the inner condyle of the humerus, commencing about two inches above its tuberosity, and extending as far down as the articulation. A second incision, of the same length, should be made along the spine, of the external condyle, and be connected with the first by a third, which should be carried transversely through the skin and the tendon of the triceps extensor cubiti, immediately above the olceranon. The quadrilateral flap thus formed is to be raised from below upwards, and held out of the way by an assistant.

The soft parts which adhere to the anterior part of the humerus, opposite the point where we wish to apply the saw, should then be detached and protected from injury by passing beneath the bone the handle of a scalpel; after which the diseased part should be carefully sawed off and the articulation destroyed. We then pass to the bones of the fore-arm.

The external lateral incision should be continued along the radius as far as necessary; and after protecting the soft parts adjacent to the head of that bone, it should be excised, endeavoring, at the same time, to preserve, as far as possible, all that portion which gives attachment to the biceps flexor cubiti. We should then lay bare the ulna, by prolonging the internal lateral incision downwards. The flap which results should be turned down, the soft part protected; and the bone divided by the application of the saw.

The tourniquet should now be relaxed; the arteries secured; the lips of the flaps brought in contact by a few sutures, and the limb placed in a state of semi-flexion.

In order to avoid injuring the ulnar nerve, Dupuytren recommends dividing the sheath in which it is enveloped, and bringing it in front of the internal condyle of the humerus, where it is to be held by an assistant, during the section of that bone.

Others have advised making the two flaps first, and even excising the bones of the fore-arm without disarticulating the fragment of the humerus. It is preferable, however, to pursue the method of Moreau, in order to ascertain by inspection of the articular surface, whether it be necessary to excise these two bones, and the extent of the incision required.

### IX.—Excision of the scapula-humeral articulation.

Several different methods have been proposed for this operation, all of which may be reduced to two principal ones, the simple incision and the formation of a flap.

The former is more particularly applicable in cases of comminuted fractures of the humerus, in consequence of gunshot wounds, where the simple enlargement of the wound does not admit of the extraction of all the osseus fragments.

The latter or the flap opertion, is then generally preferable. M. Moreau makes a quadrilateral flap with its base downwards; Manne, with its base upwards; Sabatier, a triangular flap with its base above; and Monel, a semi-lunar flap with a superior base. Mr. Syme makes first a longitudinal incision of four inches in length on the middle of the deltoid muscle, and afterwards a shorter one beginning at its inferior extremity and running up behind the posterior part of the arm-pit. All these methods could be applied here, provided we limited ourselves to the formation of a single flap.

It appears evident to us, in the first place that the joint should be attacked on the side; and in the second, that the parts should be widely opened, so that we may be ready to extend the operation, should the scapula participate in the caries. With these views the lateral and posterior flap, executed after the manner of M. Lesfranc, is incontestibly superior to all others.

Nothing can be more simple than the operation after the flap is once made. Tne next thing to be done is to cut away the synovial capsule, and after having protected the soft parts with a compress, the head of the bone should be forced outwards and sawed off. Every portion of the synovial capsule should be removed, otherwise the remaining shreds would retard the recovery of the wound.

If the caries extend to the scapula, every particle of it should be removed with the gouge and mallet.

### X.—Excision of the Clavicle.

#### 1. *Excision of the Scapular Extremity.*

This operation was practised by M. Velpeau for necrosis of the external third of the bone. He commenced by making a crucial incision, the two branches of which were four inches in length; then dissected the flaps; divided the acromio-clavicular ligaments, and the fasciæ of the deltoid and trapezius muscles; and by the aid of a piece of wood thrust in the articulation by way of a lever, raised the diseased portion of the bone and detached it from the healthy parts.

He thought that he would succeed still better, by the help of an incision parallel with the clavicle and made some lines beneath it, terminating at the acromion process and by that of another smaller one, made at a right angle with this extremity, circumscribing thus a triangular flap. The ordinary or chain saw would answer equally well in this proceeding. In all cases, where the saw is to be applied near the middle of the bone, the operation is rendered perilous, in consequence of the vicinity of the axillary vessels.

### 2. *Excision of the Sternal Extremity.*

This operation was performed in a very particular case, by Doctor Davie of England.

A young lady was affected with a distortion of the vertebral column, to such an extent, that the left scapula was forced inwards and forwards, and the clavicle, gradually obeying the impulse, quitted the cavity of the sternum and was carried back on the œsophagus, pressing upon it with such force, that deglutition could only be performed with the greatest difficulty, the life of the patient being daily threatened by the emaciation which ensued.

Every thing being prepared for the operation, Dr. Davie made on the extremity of the displaced bone, following its axis, an incision of two or three inches in length; divided the ligaments as far as the bistoury could reach; glided underneath the bone a bit of soft leather; then, with the flexible saw of Scultelus divided it about an inch from its articular surface. The bone still remaining attached to the sternum by the inter-articular ligament; it became necessary to tear this ligament away, which was accomplished by means of the handle of the bistoury, introduced between the two bones, by way of a lever, and then removed the fragment.

The wound was healed without accident; deglutition re-established; the patient was soon restored to health, and was still living six years after the operation.

### 3. *Complete extraction of the Clavicle.*

This operation was practised with entire success by Dr. Mott, of New York, in a case of osteo-sarcoma. The tnmour was as large as the two hands closed, and extended upwards nearly to the hyoid bone, and the angle of the jaw. He commenced by making, beneath the tumour, a semilunar incision, having its convexity downwards, extending from one articulation to the other; and then another from the acromion to the external side of the internal jugular vein; cut away the platysma myoid, and a portion of the trapezius muscles; passed a grooved director under the bone near the acromion; and with the chain saw completed the first section. Not being able still to remove the tumour, he joined the two incisions on the inside; applied two ligatures to the external jugular vein, and divided it in the space between them; cut away also the external portion of the mastoid muscle; tied and divided the internal jugular vein; and separated with the handle of the scalpel, the sub-clavian vein and the thoracic duct from the diseased tissues. He then returned below; divided the pectoralis major; the costo-clavicular ligament, together with the sub-clavian muscles; and terminated finally, the disarticulation of the bone, near the sternum. More than forty ligatures were applied. The wound was nearly healed in six weeks, and by means of an appropriate machine, supplying the place of the clavicle, the patient was enabled to execute nearly all the movements of the arm.

It appears to us that the method of three incisions circumscribing a quadrilateral flap, would be preferable here even if we should be obliged to cut off a portion of the integuments afterwards.

### XI.—Excision of the Scapula.

M. Janson removed a large portion of this bone on account of a tumour which occupied it.

He circumscribed the tumour by two semi-elliptical incisions, sparing the integuments as much as possible; dissected the lips of the wound; detached the tumour and the bone on all sides as far as the sub-scapular fossa; cut away the attachments of the trapezius, the supra-spinatus and the infra-spinatus muscles, and discovering that the portion of the bone beneath the spine was sound, he separated from it, with the saw the whole of the diseased portion, preserving thus the articulation of the arm. Another incision was yet necessary to enable him to expose and remove the tumour. The wound which resulted was six inches in breadth by nine in length. The patient retained the movements of the arm in the glenoid cavity.

### Art. II.—Excision of the bones of the inferior extremities.

### 1. *Excision of the anterior extremity of the first metatarsal bone.*

Excision is never practised on the phalangeal or the heads of the metatarsal bones of the four last toes; amputation is in these cases far preferable. But with regard to the first M. Blandin remarks, that when the phalanx can be saved, the foot preserves a point d'appui far more solid than it would otherwise have.

In performing the operation the surgeon should make the flap on the inside of the toe with its base posteriorly; denude the bone at the point where he wishes to divide it; apply the saw making the section perpendicularly to the axis of the bone; detach the flesh; and finish by separating it from the phalanx.

We could certainly obtain a more simple wound by means of a longitudinal incision, with a perpendicular one added to each of its extremities.

Complete extirpation has also been proposed. Already has Morean removed the entire bone in a case of caries; and M. Barbier of Valde-Grace, extracted it subsequent to luxation. Both of these operations succeeded perfectly.

### II. Extraction and excision of the bones of the tarsus.

As it is difficult to judge to what point the operation must be carried, no precise rules can be established. The method of the elder Moreau in a case of extensive caries is herewith subjoined.

There was an ulcer of an inch in diameter opposite the cuboid bone, and another between the third and fourth metatarsal bones, proceeding from an incision made some days previously on account of an abscess. On examination the director penetrated the interior of the os cuboides.

The operator made an incision on the external side of the foot, from the posterior third of the fifth metatarsal bone, to a point above the anterior apophysis of the os calcis, traversing at the same time the ulcer. The incision previously made in opening the abscess, appearing sufficient, it was only necessary to unite them to form a quadrilateral flap—this he lifted up and caused to be held out of the way by an assistant. The diseased bones were thus exposed; and it was found necessary to remove the cuboid, the third cuneiforme, the posterior extremity of the fourth metatarsal, the internal edge of the extremity of the fifth, and lastly the articular surface by which the os calcis unites itself to the cuboid. The tendon of the peroneus longus was preserved, and lay exposed at the bottom of the wound. The flap was afterwards brought down and retained by means of the suture. The extirpated bones were soon replaced by a substance which in time became ossified. As soon as circumstances would permit the patient was directed to walk freely with crutches; the foot recovered its natural form and even all its movements.

These operations are not very difficult, as the bones are easily exposed to view. The principal embarrassment is to disengage them from their neighbors; this we may accomplish by employing alternately the gouge and the bistoury.

Caries of the os calcis is more serious. If we remove its inferior face, the weight of the body would be deprived of its support, and the patient be obliged to walk on the toes, until he could accustom himself to the use of an artificial heel. If it should be necessary to destroy the attachment of the tendo-achilles, still greater inconvenience would result, and it would be preferable to amputate the limbs.

The younger Moreau excised a part of the os calcis. He excavated all the interior of the bone, taking care at the same time to preserve the insertion of the tendo-achilles. The patient soon recovered, but at first walked only on the toes.

Afterwards he extirpated the astragalus in a case of luxation of this bone across the integuments: here the state of the parts can alone guide the surgeon. After this operation the motions of the ankle are generally destroyed. He cites however a case where the movements were preserved.

### III. Excision of the tibio-tarsal articulation.

In performing this operation two incisions are to be made in the inferior part of the leg: one longitudinal, extending from the inferior part of the maleolus to within three or four inches above that eminence; the other transverse, commencing at the base of the former and extending as far as the insertion of the peroneus tertius. Having done this the operator makes two other incisions on the internal side, of which one is to be longitudinal and similar to that on the external side; the other transverse, commencing at the preceding and extending as far as the tendon of the tibialis anticus. The longitudinal incisions should extend as far as the bones, while the transverse should only include the integuments.

When the flap is dissected up, the fibula should be separated up, the fibula should be separated from the tendons by which it is surrounded, and cut away with the chisel. The same is to be done with the maleolus externus and the bones of the tarsus. When this has been accomplished, the tibia should be isolated from the soft parts, and a wooden spatula passed under its posterior surface. The surgeon then introduces the narrow blade of a saw under the anterior muscles, secures it to its handle, divides the bone from before backwards, and separates the diseased parts from the tarsus by turning the foot outwards. If a portion of the astragalus be affected, it may by this means be readily removed.

After the operation is completed, the flaps should be maintained in contact by a few sutures, and the limb should be kept perfectly at rest, by means of two lateral splinters and a foot board.

### IV.—Extraction of the fibula.

This operation was proposed by Desault, and since executed, it is said, by a patient after himself. It has also been successfully practised by M. Sentin, in the following manner:

In order to ascertain how far the malady, which he presumed to be necrosis, had extended itself; he made along the inferior part of the bone an incision of three inches in length, passing over an ulcer which was there developed. The bone being thus exposed, he perceived that the disease extended higher up; consequently he prolonged the incision as far as the superior extremity of the fibula; at which point, it appeared to be sound. Extraction was then instantly decided upon. He detached, with no little difficulty, the soft parts which were gangrenous in the asperities of the bone; and separated with a trephine, the diseased diaphysis from the sound head. Having done this, he interposed between the bone and the soft parts a narrow riband, which he gradually glided downwards to its base; separated with a curved saw all the diseased diaphysis from the external malleolus; and completed the section by the help of the gouge. It was necessary to tie a number of arteries, among them the posterior tibial; cut the external popliteal nerve, and finally, to apply the cautery to the tibia which participated slightly in the disease.

The cauterization was completed in about two months. The limb was frequently moved to prevent anchylosis, and at the expiration of four months, the patient could support himself nearly as firmly on this leg as on the other.

### V.—Excision of the Femero-tibial Articulation.

Four principal methods have been proposed for the performance of this operation.

The method of Park consisted in making a crucial incision with a transverse branch passing above the patella. It is now generally rejected.

*Method of the elder Moreau.*—This method consists in making a longitudinal incision on each side of the joint, between the vasti and flexor muscles of the leg, commencing about two inches above the condyle of the femur and terminating at the upper part of the tibia. These two incisions are to be connected by a third, which is to pass transversely below the patilla, and extend as far as the bone. The flap which is thus formed is then to be raised, the patella dissected out, and the condyles of the femur, being separated from the surrounding parts, are to be sawn through.

If the superior extremities of the bones of the leg participate in the affection, the diseased parts should be carefully excised, by prolonging the lateral incisions.

*Method of M. M. Sanson and Begin.*—They commenced by making a transverse incision below the patella, penetrating the articulation; then passed to the lateral incisions. The result is the same as that by the method of Moreau.

*Method of M. Syme.*—The leg being flexed at a right angle on the thigh, the operator makes, under the patella, a transverse incision slightly curved with its convexity inferiorly, which is to open the articulation and extend to the lateral ligaments. He then makes another above the patella, having its convexity superiorly, uniting its two extremities with those of the first: thus circumscribing this bone in an elliptical flap which he removes. Having done this, he destroys first the lateral and then the posterior ligaments; detaches the soft parts from the ends of the femur and tibia; protects them from injury by means of a compress; and saws off the ends of the bones. This is assuredly the most expeditious and the most simple method; and is that which M. Lesfranco teaches in his course.

After the operation the ends of the bones are to be placed in contact in order to obtain their consolidation; the lips of the wound united by sutures, and the limb put in a suitable apparatus. The elder Moreau uses a horizontal plane provided with sides of equal height and furnished with cushions. It is an imitation of the "*planchette hypanarthecique.*"

### VI.—Excision of the Coxo-femoral Articulation.

White was the first who proposed as well as the first who attempted the excision of the superior extremity of the femur. He removed four inches of this bone, by means of a simple longitudinal incision. The subject of the operation was a young man 14 years of age, who was still living eight years afterwards. Recently, M. Seutin removed six inches of the femur from a soldier, in consequence of a compound fracture. He differed from White by making a longitudinal incision from the iliac crest to a point three inches below the great trochanter. The patient died on the ninth day.

If the surgeon is willing to attempt the excision of this articulation, it may be preferable to make on the external side of the joint a triangular flap (Rosi,) or a semi-lunar flap, having its convexity inferiorly, extending from the iliac spine to the sciatic tuberosity (Velpeau,) or in fine any of the varieties of external flap proposed for the coxo-femoral disarticulation.

### Art. III.—Excision of the bones of the trunk.

### 1.—*Excision of the Bones of the Head.*

Some cases are reported where it was necessary to excise nearly the whole vault of the cranium; the rules do not differ from those for trepanning, aided by the action of the saw.

### II.—Excision and entire ablation of the superior maxillary bone.

Many surgeons have attempted the excision of portions of this bone, and in these cases the state of the diseased parts alone governed the operation. The idea of its complete ablation, as well as the execution was reserved for M. Gansoul of Lyons.

Before we proceed with the detail of the operation, let us examine the anatomy of parts. In examining the face of a skeleton, we find that the superior maxillary is fixed to the other bones of the face by three principal points only:

1st. By its superior apophysis and its articulation with the unguiform and ethmoid bones.

2d. By the orbitar edge of the malar bone as far as the spheno-maxillary fossa.

3d. By the articulation between the maxillary and palatal bones.

There is a fourth point of contact posteriorly with the pterygoid apophysis of the sphenoid, and with the palatine bone; but which yields easily by simply depressing the maxillary bone towards the interior of the mouth. In assailing these different points we encounter no large vessels; the trunk of the internal maxillary may be avoided without difficulty, and in all cases tied after the removal of the bone; moreover in case of unforeseen hemorrhage during the operation, we have the resource of compressing the carotid artery. As for the nerves, it will be necessary to divide but a single trunk of any importance, namely, the superior maxillary; and it is easy to make a section of this before luxating the bone, and thus prevent any injury from stretching.

Gensoul's method of operating is as follows:—The patient being seated upon a chair a little elevated, with the head thrown slightly backwards and supported on the breast of an assistant, the operator makes first a vertical incision extending from the greater angle of the eye to the upper lip, which he divides down to the level of the canine tooth; from the middle of this incision, or rather a little nearer the base of the nose, he makes a second, prolonging it to within four lines of the lobule of the ear; then a third, which descends from a point about five or six lines outside of the external orbitar angle, to the termination of the second (near the ear.) A quadrilateral flap is thus formed which he dissects up and turns back upon the forehead.

The bone being thus exposed he commences with the aid of the chisel and mallet, the section of the external orbitar angle near the suture uniting the malar bone with the external orbitar process of the frontal; and next divides the zygomatic process of the malar bone. Proceeding to the internal and superior attachment, he applies a large chisel beneath the internal angle of the eye, causing it to traverse the inferior part of the unguiforme bone and the orbitar face of the ethmoid. He separates in like manner the superior (ascending) process from the corresponding nasal bone; detaches with a bistoury the soft parts which unite the wing of the nose to the upper jaw; draws the first incisor from the bone on which he is operating and introduces a chisel into the mouth, and in the act of withdrawing it, insinuates it between the two maxillary bones, thus, easily and quickly effecting their separation. Finally he divides the superior maxillary nerve; destroys the adhesions of the pterygoid apophysis, and carries the chisel flatly between the soft parts and the orbitar plate far enough to divide completely the nerve, and find a point of support in order to depress the maxillary bone towards the mouth. Nothing remains now but to separate with a pair of curved scissors or a bistoury all the soft parts which still adhere to it, especially the curtain of the palate, so as to leave the soft portion of the taller stretched between the pterygoid process of the other side and the mouth. The wound resulting from the operation was bounded on the inside by the nasal fossae, on the outside by the cellular tissue which occurs in great quantity under the buccinator muscle, and above by the abductor muscle of the eye and the fatty tissue of the orbit; behind could be seen the posterior throat above the veil of the palate. With the maxillary was removed part of the malar, the unguiforme, ethmoid and inferior turbinated bones. The operation is much less serious than might be supposed, and is quickly performed; in one case it occupied but two minutes and a half. It is rarely necessary to tie more than one or two small arterial branches. M. Gensoul has performed this operation eight times without loosing a single patient.

The wound is suffered to drip a half hour, or hour, and the flap then united by the twisted suture.

The only difficulty attending the operation is the flow of blood into the throat; it is on this account that the patient is directed to be seated, and the operation commenced by detaching the malar bone.

The flap leaves a triple cicatrix, and is also sometimes insufficient. Would it not be practicable as indicated by M. Velpeau, to commence the incision at the labial commissure, and direct it outwards, then superiorly as far as the temporal fossa? At all events it might be necessary, the better to expose the bone, to incise vertically the superior lip as far as the nasal orifice of that side; the cicatrices would be much less disagreeable than those resulting from the incisions of M. Gonsoul.

### IV. Excision and complete ablation of the inferior maxillary bone.

The excision of this bone was performed for the first time by Dupuytren, in the year 1812. Since then it has been often practised; Walther has removed successfully the entire bone. It is conceived moreover that the progress of the disease ought to produce many differences in the mode of operating; we shall reduce them, with M. Lesfranc to the five methods which follow.

1st. *Excision limited to the centre of the body of the bone.*

*Method of Dupuytren.*—The patient being seated with his face to the light and his feet resting on a low stool, an assistant, placed behind, embraces the head with his two hands and holds it firmly against his breast; and if necessary compresses the external maxillary arteries at the base of the jaw.

The surgeon, placed before to the right of the patient, seizes with the left hand the right side of the inferior lip, at the same time that an assistant takes hold of the left side; and separates it from the superior. With a convex bistoury he divides the whole thickness of the lip, from above downwards in the median line, at first as far as the base of the jaw; then, searching with the left fore-finger for the prominence of the hyoid bone, he prolongs the incision to that point, dividing only the skin and cellular substance. Two flaps are thus formed which he dissects on each side as far as the extent of the disease may render it necessary, keeping close to the bone in order to avoid the labial arteries. These flaps being turned outwards and confided to assistants, he divides the periosteum at the point where the saw is to be applied, and having well determined the limits of the excision, extracts on each side the corresponding tooth to facilitate the action of that instrument. This being done, he passes behind the patient and applies the saw; in this position the section is easily made, whilst if he stands in front, the extremity of the saw enters the mouth and strikes against the arch of the palate, which doubles the difficulty of the operation. He protects, moreover, the nose and the upper lip by a plate of lead, a piece of pasteboard, or a thick compress. The diseased portion of the bone being sawn through, the operator returns to the front, seizes it with his left hand, and whilst an assistant holds the tongue out of the way with a spatula, passes a straight bistoury from below upwards behind the bone, detaching from left to right, the soft parts which adhere to it. The operation being finished, and the vessels tied, he brings together the flaps and retains them by means of the suture, leaving, however, a sufficient space at the inferior angle for the application of a small batch of lint and the discharge of pus. When the osseous excision makes it necessary to cut away a portion of the skin, he marks out a flap for removal by two incisions in the shape of the letter V in such manner as to join the two edges together always on the median line.

If the longitudinal incision will not suffice owing to the extent of the disease, he converts it into a crucial one, by making another along the base of the maxillary bone.

When a small extent only, of the bone is to be removed, the saw may be applied perpendicularly; but when the excision is extensive, in order to bring the fragments in apposition, it is better that the section should be made more or less oblique according to the thickness. In that case the operator begins by four or five light perpendicular strokes of the saw, and then inclines it in such manner as to gain the desired obliquity. In all cases a solid point of support may be obtained by applying the lower jaw against the upper, at least in the commencement of the section.

The submental, labial and lingual arteries giving in general but little blood; an assistant controls them with his fingers, or they are tied according as they are cut; but sometimes owing to the disease, their section causes a very abundant hemorrhage, to oppose which the actual cautery should be applied, taking care that it does not act upon the bone. If the dental artery bleeds, it should be stopped with a little piece of soft wax.

One of the most serious accidents that can happen during this operation, is the retraction of the tongue backwards, after the division of its attachments to the maxillary bone, falling into the pharynx, pressing the epiglottis down upon the larynx, and giving rise to imminent danger of suffocation. We have seen a case of this kind where it was necessary to bring the tongue forwards again with a hook. M. Lallemand was even obliged, in order to save one of his patients to practice laryngotomy.

*Method of Delpech.*—Before sawing the bone he plunges a bistoury behind it, and through the incision thus made, passes a piece of wood to protect the soft parts; another guards the tongue and superior lip. The section of the bone being made, he seizes the tongue at its point with a double hook and confides it to an assistant and not until this is done does he divide its attachments. Afterwards in using the suture provided it be the interrupted; he takes care to pass a skein of thread through the frenum of the tongue, or even through the supra-hyoid muscles, embracing likewise the cutaneous flaps; if, however, the twisted suture be employed, it should be attached to one of the pins. In one case Delpech passed through the point of the tongue a thread of gold which he fixed to the neighboring teeth: by degrees the thread cut through the portion of the tongue enclosed by it, but the adhesion which had by this time formed, was sufficient.

Finally, it is to Delpech also, that we are indebted for this important precept—to preserve one of the tables of the bone when it is sound, and to limit the excision to the other, using if necessary the rugine, the gouge or the saw. The bony table which is preserved, although it be small, affords to the soft parts a solid point of support; and gives at least to the lower jaw all the extension possible. M. Roux of Saint-Maximin, has likewise pointed out all the advantage there is in removing only half of the height of the bone, when the other half is healthy.

The differences these indications may produce in the mode of operating, are easily conceived.

*Method of M. Gensoul.*—This method rests entirely upon the form given to the exterior incision, when the extent of the osseous excision renders it necessary to cut off a portion of the skin. The ordinary v incision has the inconvenience of placing the cicatrix upon the median line ; and when the bone no longer sustains the integument of the chin, there will be a line of knotty fibrous tissue extending directly from it to the hyoid bone, which tends continually to retract, drawing the lip downwards and giving to the chin a novel appearance. Gensoul limits himself at first to the median incision; and if after accomplishing the excision he perceives that there is too much skin; he retrenches it at will, but upon one side only, in such manner as that the cicatrix may be placed laterally, and the consecutive retraction of the skin avoided.

*Relative advantages of the several Methods.*—As the retraction of the tongue is always probable, although not constant, it will be better, in order to avoid this accident, to pursue the method of Delpech. As it regards the posterior incision previous to the section of the bone, M. Lisfrance properly remarks that as the tissues adjacent to the bone, ordinarily participate in its disease, it matters but little should they be touched by the saw, since they must necessarily be removed ; moreover, in sawing with precaution, there is but little risk of tearing them. We will add that this reducing of the soft parts is much less formidable than is generally supposed, and that the earing of the inferior dental nerve, which is inevitable, leads to no accident. On this point the method of Dupuytren is simple and seems to us most preferable. For a still stronger treason we must reject another method which nothing can justify, and which consists in completely dividing the attachments of the tongue before making the section of the bone.

With respect to the position of the cicatrix, we apprehend no inconvience from the process of Gensoul ; perhaps, also, if experience confirms its advantages, it might be established as a general rule, and the simple incision made thereafter always a little to one side.

The twisted suture is generally preferred, nevertheless the interrupted suture succeeded very well in the hands of Delpech. We could also, without doubt, fix one of the fragments to the other by tying with a thread of gold the corresponding teeth; but in general it is not necessary.

2. *Excision of all the Horizontal Portion.*

*First Method—by a superior Flap.*—An incision should be made along the base of the jaw, passing from one to two lines beyond its angles; a large flap should be dissected from below upwards and turned back upon the face; the bone sawed on each side beyond the limits of the disease; then the soft parts which are inserted into it posteriorly, separated according to the principles above indicated.

If the disease extend high up on the rami of the jaw, two incisions might be made along their posterior edges, which should intersect the extremities of the first.

*Second Method by two lateral Flaps.*—A horizontal incision being made, (as above,) is to be converted into a T incision, by means of a vertical one dividing all the lower lip on the median line. This mode is easier than the first.

3. *Excision of half the Horizontal Portion.*

*First Method—inferior Flap.*—(J. Cloquet.)—With the bistoury or the chisels of Dubois a horizontal incision is made, beginning at the labial commissure and ending one or two lines beyond the ramus of the jaw. To this first incision two other vertical ones are joined, one descending from the free border of the lip to the base of the jaw, the other parallel, descending behind the ramus of the bone some lines below the angle. The flap is dissected from above downwards; the soft parts separated from the internal face of the bone, previous to making its section, which is accomplished with phalangeal, the saw *en crete de cog.*, or the chain saw.

*Second Method—two Flaps.*—(V. Mott.)—The surgeon makes with its convexity below and behind a curved incision extending from the front of the ear on a level with the condyle, nearly to the chin below the labial commissure: the superior flap is dissected up and turned back on the face. Then a second incision is to be made, descending from the superior extremity of the first to the angle of the jaw, which gives the inferior flap. The bone is divided first before, then behind, as high as is necessary and the diseased portion removed. When we go to a certain height, Mott advises the division of the inferior maxillary nerve, before exercising any traction on the separated portion of the bone, and to bear in mind the proximity of the lingual nerve, in order to avoid injuring it.

Prior to this operation the American surgeons believed it necessary to tie the carotid artery.

*Third Method—superio r external Flap.*—(M. Lisfranc.)—A vertical incision is first made, commencing at the free border of the lip and extending down under the chin; then a second, which extends from the first, along the base of the bone to a point about two lines beyond its angle. The flap is dissected from below upwards above the tumour and turned back on the face. The ramus of the bone is divided with the saw *en crete de cog.*, and the second section made near the chin with an ordinary fine saw; the tumour being removed, the flap is brought down and retained by the suture. This method, besides, being more simple than the former two, is not liable to collections of pus at the base of the flap, and leaves, finally, but a slight cicatrix on the face.

*Fourth Method—superior internal Flap.*—If the disease should extend farther posteriorly than anteriorly, we might succeed well by means of a semi-lunar incision made along the ramus and base of the jaw, extending from the ear to the chin. We would thus obtain a superior and internal flap, and avoid a cicatrix on the face after recovery.

In all these dissections we should include with the skin, the cutaneous muscles only. The masseter should be removed to the same extent as the bone. The external maxillary is always encountered, but may be easily tied before dividing it.

4. *Excision of half the bone including the articulation.*

This operation has been practised with more or less success, and in most of the cases surgeons have found it proper to tie the trunk of the carotid, before beginning the operation. We have to divide the external and internal maxillary, dental, transverse facial, *masseter* and temporal arteries and run the risk of touching the internal carotid. However, Lesfranc and others have accomplished the operation without ligatures.

*Method of Operating.*—The operator should commence by making a horizontal incision along the base of the bone, interesecting it by a vertical one which divides the inferior lip; then a third should be made extending from the first to the zygomatic arch and passing behind the ramus of the jaw; then the quadrilateral flap circumscribed by these incisions, should be dissected up and the saw applied to the bone, commencing in front. After which the soft parts should be detached from the internal face of the bone; and the tendon of the temporal muscle divided from within outwards by gliding a concave bistoury behind the coronoid process. Nothing further will remain but to detach the condyle, which is to be effected by dividing first the external articular ligament; then rotating the bone in order to stretch in turn every point of the capsule, dividing the latter with the chisels. The condyle being detatched and carried outwards, it will be easy to pass behind it a probe-pointed bistoury in order to cut away, the external pterygoid muscle.

This operation is tedious and difficult; for this reason it is necessary to tie all the arteries as fast as they are divided, and if the ligature of the carotidian trunk has been omitted, it must be kept out of the way of the knife, by an assistant.

After this, as well as after the preceding operations, the deviation of the remaining half of the bone is towards the opposite side is almost inevitable.

### 5. *Complete Ablation of the Bone.*

This operation has been performed once by Naltar of Bonn with complete success. The method to be pursued is as follows:—A horizontal incision should be made along the base of the jaw, each extremity of which should be intersected by a perpendicular one coming from each zygomatic arch, the immense flap which results is to be turned back on the face, and the operation continued first on one side then on the other, according to the directions already laid down.

It is evident that we would facilitate our object if we first sawed the bone at the middle part; by this means reducing the operation to two excisions, nor will it be necessary in order to accomplish this, to form a cutaneous on the median side

### V.—Excision of the ribs.

This operation was known for a long time before M. Richerand claimed to be the inventor.

The diseased portions of the bone should be exposed by means of a straight, curved or crucial incision, or by making at once a quadrilateral flap; after which the intercostal muscles which adhere to the rib, both above and below should be divided, either from within outwards or *vice versa.* The rib is then to be detatched from the pleura with the handle of a scalpel, and excised by means of a saw *in crete de cog.*, or by gliding behind it the chain saw.

The pleura is generally thick, and beneath the ribs, and moreover adheres to the pulmonary layer, in such manner that the introduction of air is little to be feared; sometimes, however, it is nearly sound, or again its thickness is of such a nature, that its excision becomes necessary. To effect this we may use the sharp edge of the curved chisels; the air which rushes quickly into the cavity of the thorax threatens immediate suffocation; and to prevent this the flap should be hastily brought down, or the wound completely covered with a large compress spread with cerate, after which we must be guided by the rules established on this subject for the operation of empyema.

M. Richerand further indicated that a dangerous hemorrhage from the intercostal artery at the moment of its division was greatly to be feared; this artery is of a very small calibre as it passes from the posterior third of the rib, consequently this fear is nearly without foundation.

### VI.—Excision of the sternum.

Galen is said to have removed a large portion of this bone; and several operations of the same nature have succeeded in the hands of modern surgeons. In these cases the trepan and the circular knife are generally employed: if these will not suffice we may resort to the saw *en crete de cog.*, the gouge and the mallet or even the chain saw to divide the costal cartilages, by which means we will easily terminate the operation.

### VII.—Excision of the spinous processes of the vertebræ.

This operation was performed in 1829 by Dr. Alban G. Smith of Kentucky.

Owing to a fall from a horse, a young man had the spinous process of one of the lumbar vertebræ thrown to the right about three lines from its natural position. It was supposed to be a luxation; the patient however survived; and being affected two years afterwards with paralysis of the inferior members, was willing to submit to any means for the sake of recovery. An examination was made by Dr. Dudley in the presence of Dr. Smith, and the operation decided upon.

An incision of five to six inches was made along the spinous process of the vertebræ, and to each of its extremities a transverse one was added of three and a quarter inches in length, and extending down to the bone. The muscles were then detatched from within outwards, in the two vertebral grooves, as far as the rough turbercle of the transverse apophysis, and by this means the whole deformity exposed. Four of the vertebræ participated in the fracture. With the little saw of Hey, the section of the vertebræ was made in such manner as to remove the spinous processes—the lips of the wound were then brought together, which completed the operation.

The patient, some time after, was in a fair way for recovery—the paralysis was diminished.

### VIII.—Excision of the bones of the pelvis.

In caries or osteo-sarcoma, portions of the crest of the ilium, of the ascending branch of the ischium, and the entire coxcyx have been removed. In the management of these cases one must be governed by the extent of the disease and the general rules indicated for the treatment of caries.

